# tRF-Val-CAC-016 modulates the transduction of *CACNA1d*-mediated MAPK signaling pathways to suppress the proliferation of gastric carcinoma

**DOI:** 10.1186/s12964-022-00857-9

**Published:** 2022-05-19

**Authors:** Weiguo Xu, Junyu Zheng, Xiao Wang, Bin Zhou, Huanqiu Chen, Gang Li, Feng Yan

**Affiliations:** 1 Department of General Surgery, Jiangsu Cancer Hospital & The Affiliated Cancer Hospital of Nanjing Medical University & Jiangsu Institute of Cancer Research, Nanjing, China; 2 Department of Clinical Laboratory, Jiangsu Cancer Hospital & The Affiliated Cancer Hospital of Nanjing Medical University & Jiangsu Institute of Cancer Research, Baiziting No. 42, Nanjing, 210009 Jiangsu China; 3 Department of Radiology, Jiangsu Cancer Hospital & The Affiliated Cancer Hospital of Nanjing Medical University & Jiangsu Institute of Cancer Research, Nanjing, China; 4 Department of Gastric Surgery, Jiangsu Cancer Hospital & The Affiliated Cancer Hospital of Nanjing Medical University & Jiangsu Institute of Cancer Research, Baiziting No. 42, Nanjing, 210009 Jiangsu China

**Keywords:** tRNA derivative, tsRNA, Immunoprecipitation, Hierarchical clustering, Post-transcriptional regulation

## Abstract

**Background:**

As a new kind of non-coding RNAs (ncRNAs), tRNA derivatives play an important role in gastric carcinoma (GC). Nevertheless, the underlying mechanism tRNA derivatives were involved in was rarely illustrated.

**Methods:**

We screened out the tRNA derivative, tRF-Val-CAC-016, based on the tsRNA sequencing and demonstrated the effect tRF-Val-CAC-016 exerted on GC proliferation in vitro and in vivo. We applied Dual-luciferase reporter assay, RIP assay, and bioinformatic analysis to discover the downstream target of tRF-Val-CAC-016. Then *CACNA1d* was selected, and the oncogenic characteristics were verified*.* Subsequently, we detected the possible regulation of the canonical MAPK signaling pathway to further explore the downstream mechanism of tRF-Val-CAC-016.

**Results:**

As a result, we found that tRF-Val-CAC-016 was low-expressed in GC, and upregulation of tRF-Val-CAC-016 could significantly suppress the proliferation of GC cell lines. Meanwhile, tRF-Val-CAC-016 regulated the canonical MAPK signaling pathway by targeting *CACNA1d.*

**Conclusions:**

tRF-Val-CAC-016 modulates the transduction of *CACNA1d*-mediated MAPK signaling pathways to suppress the proliferation of gastric carcinoma. This study discussed the function and mechanism of tRF-Val-CAC-016 in GC for the first time. The pioneering work has contributed to our present understanding of tRNA derivative, which might provide an alternative mean for the targeted therapy of GC.

**Video abstract**

**Supplementary Information:**

The online version contains supplementary material available at 10.1186/s12964-022-00857-9.

## Background

More than 70% of new cases of gastric cancer (GC) occurred in developing countries, and about 50% of GC cases occurred in eastern Asia, mainly in China. The number of incidences and deaths of gastric cancer in China accounted for 42.6% and 45.0% of the global statistical data, respectively, ranking 5th in morbidity and 6th in mortality among 183 countries globally [1]. Therefore, it is essential to explore the treatment approaches for GC.

tsRNAs, as a new kind of discovered ncRNAs, have attracted increasing attention in cancer research. tsRNAs were classified into tRFs and tiRNAs based on the cleavage sites of tRNAs by the ribonucleases such as Dicer and RNase Z [2, 3]. Meanwhile, with the illustration for the fundamental structures of tRFs and tiRNAs, it aroused great interest of many researchers in the aspect of biological characteristics and potential therapeutic strategies of tsRNAs in several kinds of carcinomas [4–8]. tsRNAs have been demonstrated as mRNA regulators by binding to the 3′UTR of specific mRNAs, similar to miRNAs [9].

*CACNA1d* has been verified as an oncogene in several articles [10–12]. It was involved in the MAPK signaling pathways to regulate the proliferation of certain carcinomas [13]. As one of the calcium voltage-gated channel subunits, *CACNA1d* also played a role in the modulation of calcium ion concentration inside and outside the cells [14]. Furthermore, the MAPK signaling pathways was widely accepted as the canonical pathway to regulate the progression of GC in many types of studies [15–19]. The bioinformatic analysis indicated that *CACNA1d* was located in the cell membrane and participated in the MAPK signaling pathways, consistent with a few previous studies [13, 20].

Concerning the present study, we screened out the tRF-Val-CAC-016 as the research target, and it was significantly down-regulated in GC tissues. Furthermore, we found MAPK signaling pathways and *CACNA1d* were regulated by tRF-Val-CAC-016. Therefore, we hypothesize that tRF-Val-CAC-016 modulates the transduction of *CACNA1d*-mediated MAPK signaling pathways to suppress the proliferation of gastric carcinoma.

## Methods

### Clinical samples

All GC samples were collected in Jiangsu Cancer Hospital with informed consents from these patients. Meanwhile, this study was appraised and authorized by the ethics committee of Jiangsu Cancer Hospital (NYDLS-2019-919). In this study, we classifed and analyzed the collected GC samples from 40 patients according to the data such as age, gender, tumor size, histology, and stage. We grouped the age and tumor size with median and mean, respectively. The eighth edition American Joint Committee on Cancer (AJCC) was applied to implement the classification of TNM stage. Details were presented in Table 1. All samples were snap-frozen in the refrigerator at − 80 °C, and the Declaration of Helsinki was obeyed.

**Table 1:** Pathological association between the tRF-Val-CAC-016 expression and the clinicopathological features in 40 pairs of GC tissues

Characteristics		Case	tRF-Val-CAC-016 expression		*p*-value
			Low	High	
All		40	25	15	
Age (years)	< 63	15	9	6	0.800
	≥ 63	25	16	9	
Gender	Male	33	22	11	0.237
	Female	7	3	4	
Size (cm)	< 6.0	21	9	12	0.007*
	≥ 6.0	19	16	3	
Histology	Well-differentiated	16	6	10	0.008*
	Poor-differentiated	24	19	5	
TNM stage	I–II	10	6	4	0.850
	III–IV	30	19	11	

^*^p < 0.01

### tRFs and tiRNAs sequencing profiles

The purity and concentration of total RNA samples were determined with NanoDrop ND-1000. Total RNA samples are pretreated to remove some RNA modifications that interfere with small RNA-seq library construction. Subsequently, total RNA of each sample was sequentially ligated to 3′ and 5′ small RNA adapters. cDNA was then synthesized and amplified using Illumina’s proprietary RT primers and amplification primers. Subsequently, ~ 134–160 bp PCR amplified fragments were extracted and purified from the PAGE gel. Finally, the completed libraries were quantified by Agilent 2100 Bioanalyzer. The libraries were denatured and diluted and were then loaded onto reagent cartridge and forwarded to sequencing run on Illumina NextSeq 500 system using NextSeq 500/550 V2 kit (#FC-404-2005, Illumina), according to the manufacturer’s instructions. We then screened out the tRFs and tiRNAs based on the fold-change (log_2_FC ≥ 1 or log_2_FC ≤ − 1) of expression data and *p*-value (*p* < 0.05) of differentially expressed tsRNAs. Hierarchical clustering, Volcano plots, and Correlation Analysis were then used to present the essential data. GO and KEGG analysis was then conducted to sieve the significant target genes and signaling pathways. Subsequently, we applied the Gene Set Enrichment Analysis (GSEA) further to illustrate the potential characteristics of selected target genes. In addition, to comprehensively verify the expression levels and prognostic data of selected genes, we downloaded the GC-related data in TCGA (TCGA-STAD) and GEO databases (GSE65801). Finally, we imported all bioinformatic data into R software to select and verify the target genes.

### Cell culture and transfection

We obtained GC cell lines NCI-N87, HGC-27, SNU-216, BGC-823, AGS, and GES-1 from the Cell Bank of the Chinese Academy of Sciences (Shanghai, China), and were authenticated to be free from mycoplasma infections. NCI-N87 was cultured in the RPMI-1640 medium with 15% FBS, HGC-27, SNU-216, BGC-823, and GES-1 in the RPMI-1640 medium 10% FBS, AGS was cultivated in the F-12K medium with 10% FBS. All cell lines were incubated in a humidified incubator at 37 °C with 5% CO2. We complied with the transfection with lipofectamine2000 (Invitrogen, USA) following the manufacturer’s instructions. Subsequently, we applied p38 MAPK-IN (MCE, USA) as the inhibitor of the MAPK signaling pathway.

### RNA isolation, reverse transcription, and qPCR

Total RNA was extracted from GC cell lines and tissue samples with Trizol reagent (Life Technologies, USA), then the reverse transcription (RT) for tsRNAs was performed with the riboSCRIPT Reverse Transcription Kit (RiboBio, Guangzhou, China), and the manufacturer’s protocols were followed (60 min at 42 °C and 10 min at 70 °C). We operated RT for *CACNA1d* using PrimeScript™ RT Master Mix (Takara, Japan) following the manufacturer’s instructions (15 min at 37 °C and 5 s at 85 °C). Finally, we applied LightCycler 1.5 (Roche, Switzerland) to conduct qPCR in a 20 μL reaction system (2 μL cDNA, 6.4 μL DEPC, 10 μL SYBR Green Mix, 0.8 μL forward primer, and 0.8 μL reverse primer). qPCR reaction conditions were 95 °C for 30 s, 40 cycles of 95 °C for 5 s and 60 °C for 20 s. GAPDH served as an internal control for *CACNA1d*, and U6 was used as an internal control for tsRNAs.

### Fluorescent in situ hybridization (FISH)

Cell fixation was performed with 4% paraformaldehyde, and the fixed cells were then permeabilized for 5 min at 4 °C with Triton X-100 (Beyotime, Shanghai, China). Subsequently, pre-hybridization, hybridization, and blocking were undertaken with the Ribo™ Fluorescent In Situ Hybridization Kit (RiboBio, Guangzhou, China). Finally, the cells nuclei were stained blue with 4′, 6-diamidino-2-phenylindole (DAPI), and cytoplasm was stained red with the Cy3-labelled tRF-Val-CAC-016 (GenePharma, Shanghai, China).

### Cell proliferation assays

In the CCK-8 assay, 100 μL cell suspension containing 1000 cells was seeded in each well of 96-well plates, then 10 μL CCK-8 reagent (Dojindo, Laboratories, Japan) was added into each well and incubated for two hours. We then measured the OD value in 450 nm by the microplate reader at 0, 24, 48, 72, and 96 h. In the cell colony formation assay, 2 ml cell suspension containing 4000 processed cells were seeded into each well of 6-well plates. After the incubation for 10–14 days, the cells were fixed with 4% paraformaldehyde for 20 min at room temperature (RT) and were then stained with 1% crystal violet. Images were captured with a microscope, and the number of colonies was counted with Image J software.

### Cell cycle determination

We harvested the transfected cells and resuspended them in 70% ethanol. Afterward, cells were centrifuged, and the cell pellets were resuspended in PI-solution to conduct the flow cytometry analysis.

### EdU assay

Ethynyl-2′-deoxyuridine (EdU) assay was undertaken with the Cell-Light EdU Apollo In Vitro Kit (Ribobio, Guangzhou, China). Each well of 96-well plates was seeded with 4 × 10^3^–1.0 × 10^5^ cells, and these GC cells were then incubated in the EdU solution (50 μM) for two hours. Subsequently, the cell fixation was performed with 4% paraformaldehyde, and cells were then washed with 50 μL glycine (2 mg/mL). Analogously, we added 100 μL penetrant (0.5% TritonX-100) into each well to incubate these cells for 10 min. Next, we pipetted 1 × Apollo reagent (100 μL) into each well to initiate the staining reaction. Nuclei were stained with DAPI or 1 × Hoechst33342, and images were captured with a fluorescence microscope.

### Dual-luciferase reporter assay

GC cells were co-transfected with plasmids WT-CACNA1d-3′UTR or MUT-CACNA1d-3′UTR, and tRF-Val-CAC-016 mimics or mimic control with lipofectamine2000 (Invitrogen, USA). We detected the luciferase activities of firefly and renilla with the Dual-Luciferase Reporter Assay System (Promega, Madison, USA). To begin with, we obtained the cell lysates using the Passive Lysis Buffer. After preparing Luciferase Assay Reagent II (LAR II) and Stop & Glo Reagent, we carefully transferred 20 µl of cell lysate into the luminometer tube containing LAR II to record the firefly luciferase activity measurement. Then we added 100 µl of Stop & Glo Reagent to record the renilla luciferase activity measurement.

### RNA-binding protein immunoprecipitation (RIP)

We purchased the Magna RIP™ RNA-Binding Protein Immunoprecipitation Kit (Merck, Darmstadt, Germany). First, we lysed GC cells in RIP lysis buffer (0.5 M Tris–HCl, pH 7.4, 1.5 M NaCl, 2.5% deoxycholic acid, 10% NP-40, 10 mM EDTA) and dispensed 200 μL each of the lysates into nuclease-free microcentrifuge tubes and stored them at − 80 °C. Second, we incubated the washed beads with Argonaute-2 antibody to produce the beads-antibody complex and used the Normal Rabbit IgG antibody as the negative control. Third, 100 µL supernatant of the RIP lysate was removed and added to each beads-antibody complex in RIP Immunoprecipitation Buffer (10 µL of the supernatant was stored at − 80 °C as Input). All the tubes were incubated and rotated overnight at 4 °C. Finally, RNA purification and RT-PCR were manipulated to test the binding tRF-Val-CAC-016.

### Immunohistochemistry (IHC) and immunofluorescence staining

Tissue sections were deparaffinized and dehydrated, and then the tissues were blocked with serum and placed in the 37 °C incubator for half an hour. Next, We added the primary antibodies to the slides and incubated them overnight in the 4 °C incubator. Analogously, we then added the secondary antibodies to the sections and set them at 37 °C for half an hour. Afterward, we stained these tissue sections with DAB substrate and hematoxylin successively and dehydrated them with ethanol. Images were captured with a microscope. In terms of immunofluorescence staining, cells were rinsed and rewarmed with PBS for 20 min. We then applied 0.5% TritonX-100 to permeabilize the sections at RT for 20 min. Subsequently, serum blocking and antibody incubation were performed successively. Nuclei were stained with DAPI. We collected images using a fluorescence microscope.

### Subcutaneous xenograft experiments

Eighteen five-week-old Balb/c female nude mice were purchased from the Shanghai Experimental Animal Center of the Chinese Academic of Sciences (Shanghai, China) and were divided into tRF-Val-CAC-016 agomir group, tRF-Val-CAC-016 negative control (NC) group, and normal saline (NS) group randomly and evenly. These reagents were purchased from Ribobio (Ribobio, Guangzhou, China). Approximately 1.2 × 10^6^ processed NCI-N87 cells were then subcutaneously injected into the right flanks of these mice. Tumor sizes and weights were measured every 3 days. After 39 days of rearing, all mice were sacrificed, and all tumors were resected. Tumor volumes were calculated with this formula: 1/2 × (length × width^2^). All animal experiments complied with the National Research Council’s Guide for the Care and Use of Laboratory Animals. The care of animals was in accordance with the institution guidelines of Jiangsu Cancer Hospital.

### Immunoblotting

Gel-electrophoresis (10% SDS-PAGE) for extracted proteins was conducted, and the proteins were then transferred to a polyvinylidene difluoride membrane. Next, we blocked the membranes with 1 × Tris-buffered saline (TBS) containing 0.1% Tween 20 and 5% BSA or 5% skim milk for one hour at RT. Then, we incubated the membranes with specific primary antibodies overnight at 4 °C and the secondary antibodies for two hours at RT. After three washes, the membranes were visualized with the chemiluminescence (ECL) kit (Millipore, Bedford, MA, USA). Basic information on the antibodies used in this research is presented in Additional file 1: Table S1.

### Statistical Analysis

We utilized GraphPad Prism Version 4 program (GraphPad Software Inc., San Diego, CA) and SPSS 19.0 (IBM, Chicago, USA) to deal with the data. All numeric values were presented as the mean ± SD. The significant data was determined using ANOVA followed by Dunnett’s test or Student’s unpaired t-test. *P* < 0.05 was taken as statistically significant.

## Results

### tRFs and tiRNAs sequencing profiles of GC tissues

**Fig. 1:**
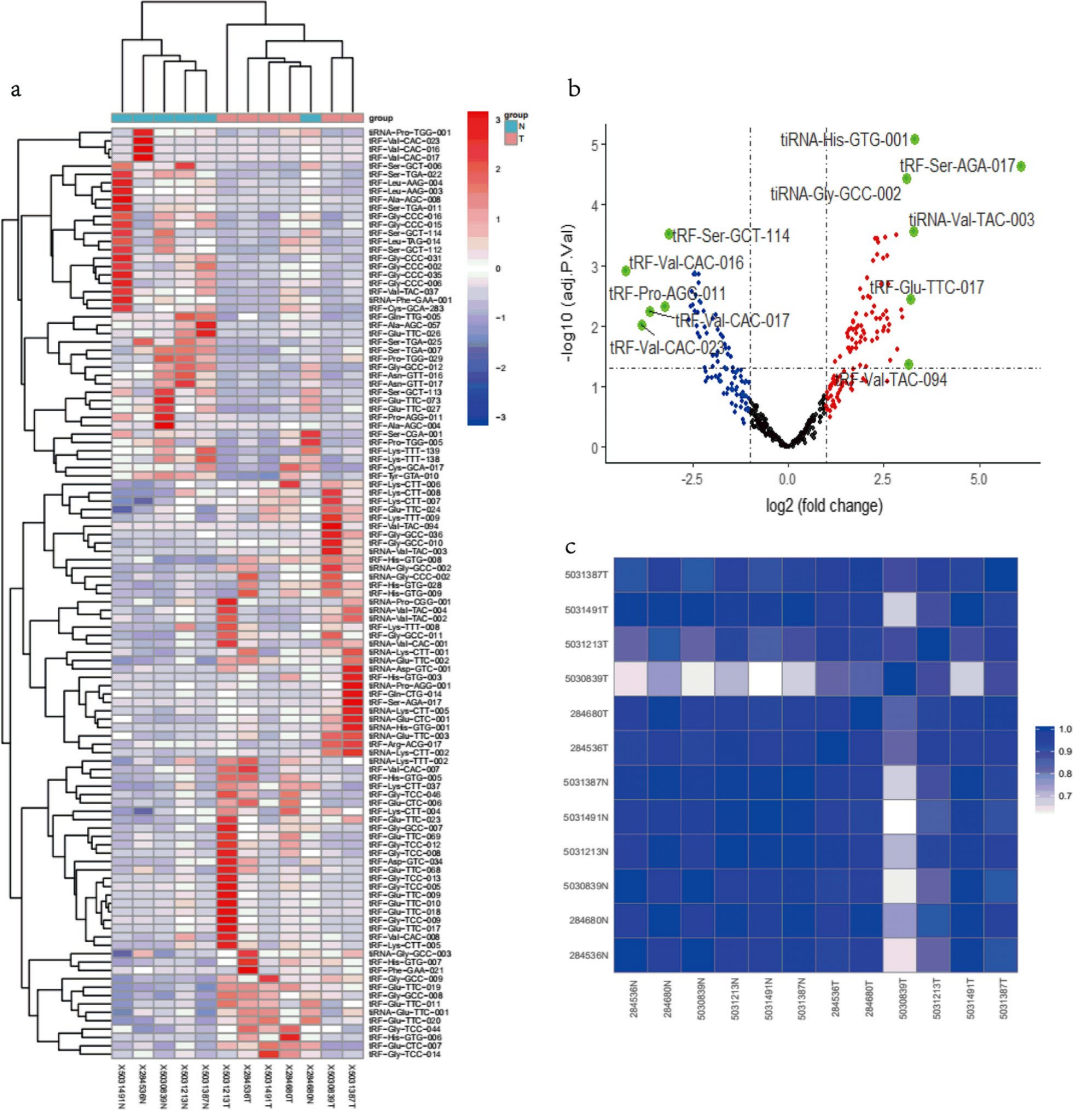
tRFs and tiRNAs sequencing profiles of GC tissues. **a** Heatmap of the differentially expressed tsRNAs (DETs). **b** A total of 69 up-regulated and 42 down-regulated DETs and 277 NDETs were presented using the volcano plot, and five down-regulated and six up-regulated tsRNAs were obviously shown in the plot based on the foldchange (FC) of the profiles (log_2_FC ≥ 3 or log_2_FC ≤ − 3, *p* < 0.05). **c** Heatmap of the correlation coefficient. The color in the panel represents the correlation coefficient of the two samples. Blue represents a high correlation coefficient between the two samples, and white indicates the low similarity of the two samples

We performed tsRNAs sequencing to distinguish the differentially expressed tsRNAs (DETs) and not differentially expressed tsRNAs (NDETs), and tRF-Val-CAC-016 was finally selected based on the profiles. Subsequently, we applied the hierarchical clustering to classify the DETs, a total of 69 up-regulated and 42 down-regulated DETs were presented with hierarchical clustering heatmap (Fig. 1a). We used the volcano plot to exhibit the most discrepant DETs, which indicated that five down-regulated and six up-regulated tsRNAs were presented in the plot based on the foldchange (FC) of the profiles (log_2_FC ≥ 3 or log_2_FC ≤ -3, *p* < 0.05) (Fig. 1b). Obviously, we selected tRF-Val-CAC-016 for further research considering the feasibility and statistical significance. Meanwhile, we accomplished the heatmap of the correlation coefficient to elaborate on the similarity of the GC samples using the R gplots package (Fig. 1c). The distribution and frequency of tsRNA subtypes were presented in Additional file 2: Fig. S1a–b and c–d, respectively. The classification of tsRNA isodecoders is shown in Additional file 2: Fig. S1e–f.

### tRF-Val-CAC-016 was significantly low-expressed in GC tissues

**Fig. 2:**
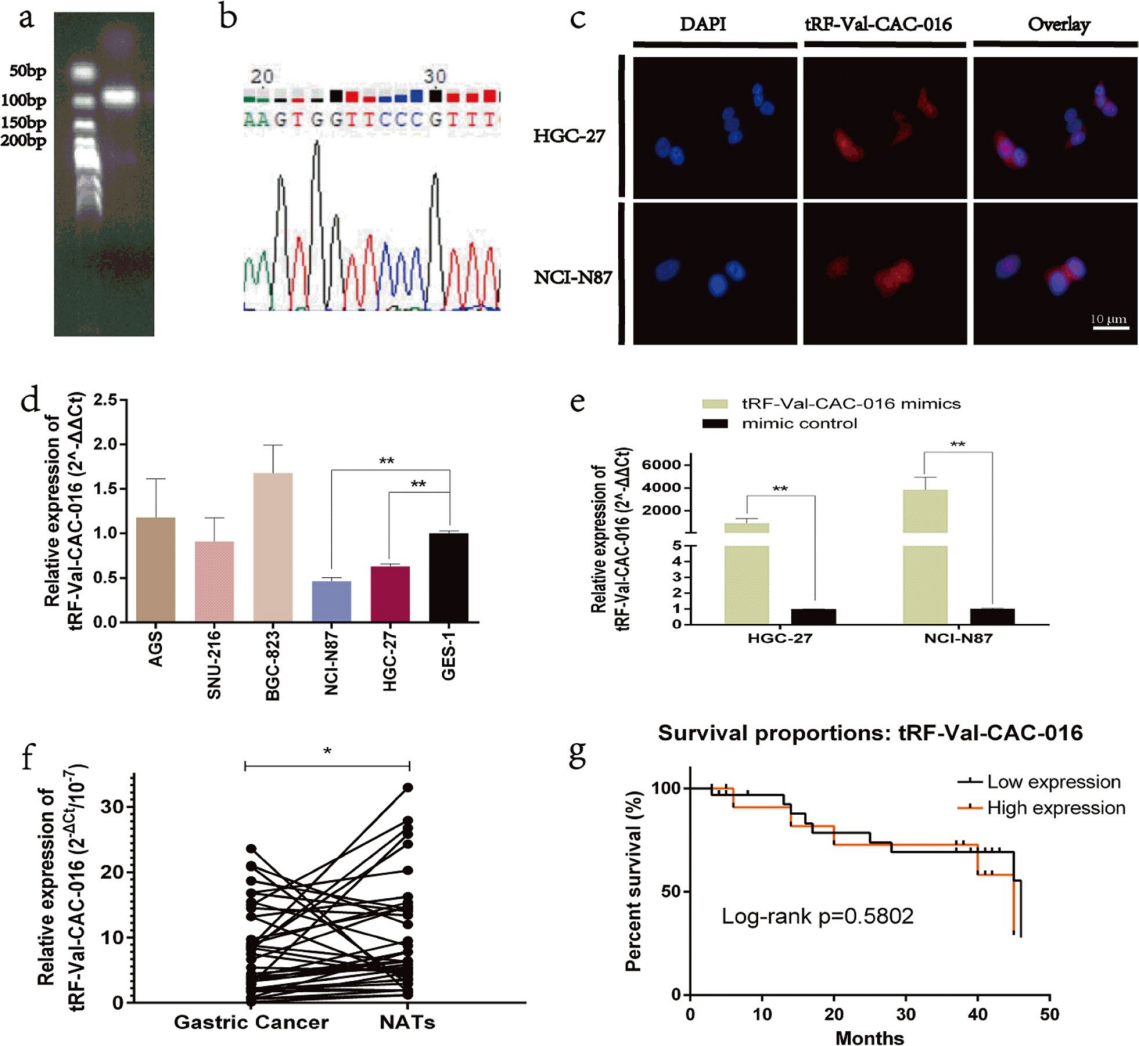
tRF-Val-CAC-016 was significantly low-expressed in GC tissues. **a**–**b** Gel-electrophoresis was applied to verify the PCR product of tRF-Val-CAC-016, and the Sanger sequencing was undertaken to confirm the results. **c** Fluorescent In Situ Hybridization (FISH) assay indicated that tRF-Val-CAC-016 is located in both nuclei and cytoplasm, and mainly in the cytoplasm. **d** The expression levels of tRF-Val-CAC-016 were tested in GC cell lines, NCI-N87 and HGC-27 were finally selected. **e** tRF-Val-CAC-016 mimics could significantly increase the expression level of tRF-Val-CAC-016. **f** The low expression level of tRF-Val-CAC-016 was confirmed in 40 pairs of GC tissues. **g** Correlation between the expression level of tRF-Val-CAC-016 and the long-term outcomes (overall survival, OS) of GC was not significant (*P* = 0.5802). *P < 0.05, **P < 0.01, statistically significant. Scale bar = 10 µm

We applied Gel-electrophoresis to verify the PCR product of tRF-Val-CAC-016, demonstrate the feasibility of PCR primers, and verify the authenticity of tsRNA sequencing (Fig. 2a). As shown in Fig. 2b, the length of tRF-Val-CAC-016 ranged between 50 and 100 bp, and we undertook the Sanger sequencing to verify the results. Then Fluorescent In Situ Hybridization (FISH) assay indicated that tRF-Val-CAC-016 is located in both nuclei and cytoplasm, but mainly in the cytoplasm (Fig. 2c). Afterward, the expression level of tRF-Val-CAC-016 was tested in GC cell lines, NCI-N87 and HGC-27 were finally selected (Fig. 2d). Finally, to confirm the efficiency of tRF-Val-CAC-016 mimics, we conducted the transfection, and the result was in line with our expectations (Fig. 2e). Analogously, the low expression level was confirmed in 40 pairs of GC tissues (Fig. 2f). Meanwhile, the expression of tRF-Val-CAC-016 was significantly associated with tumor size and histology in the aspect of clinicopathological features (Table 1). Furthermore, the prognostic outcome of tRF-Val-CAC-016 was calculated after the follow-up for the patients after gastrectomies, but the result was not significant (Fig. 2g). The sequences of tRF-Val-CAC-016 and related primers are listed in Additional file 3: Table S2.

### tRF-Val-CAC-016 suppressed the proliferation of GC

**Fig. 3:**
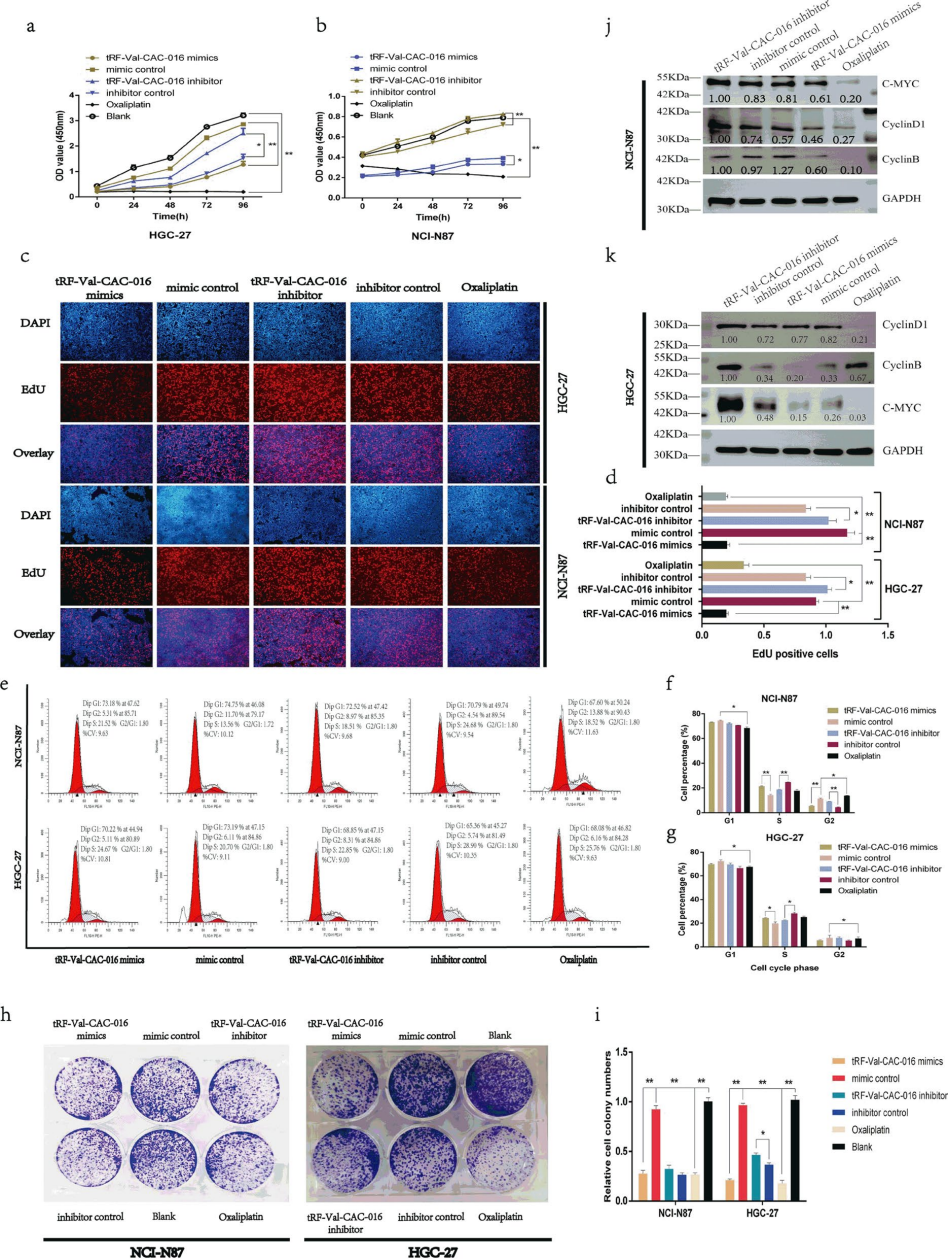
tRF-Val-CAC-016 suppressed the proliferation of GC. **a**–**b** CCK-8 assays for tRF-Val-CAC-016 mimics, tRF-Val-CAC-016 inhibitor, and oxaliplatin. **c–d** The ethynyl-2′-deoxyuridine (EdU) assays demonstrated that tRF-Val-CAC-016 could suppress the cell replication activity, but tRF-Val-CAC-016 inhibitor enhanced the replication activity of GC. **e–g** tRF-Val-CAC-016 adjusted the S phase significantly both in NCI-N87 and HGC-27. **h–i** tRF-Val-CAC-016 modulated the viability of GC cells in colony formation assays. **j–k** Immunoblotting assays for CyclinD1, CyclinB, c-myc. *P < 0.05, **P < 0.01, statistically significant

As shown in Fig. 3a–b, tRF-Val-CAC-016 significantly suppressed the proliferation of GC cells in CCK-8 assays, achieving a similar performance of oxaliplatin. Besides, tRF-Val-CAC-016 inhibitor promoted the proliferation of GC compared with the inhibitor control. The ethynyl-2′-deoxyuridine (EdU) assays demonstrated that tRF-Val-CAC-016 could suppress the cell replication activity of GC, but tRF-Val-CAC-016 inhibitor enhanced the replication activity (Fig. 3c–d). Meanwhile, we found that tRF-Val-CAC-016 could regulate the checkpoints of the cell cycle in GC. As presented in Fig. 3e–g, oxaliplatin mainly handled the G1 phase in GC. However, tRF-Val-CAC-016 adjusted the S phase significantly both in NCI-N87 and HGC-27. In colony formation assays, tRF-Val-CAC-016 could inhibit the viability of GC cells, slightly weaker than oxaliplatin. tRF-Val-CAC-016 inhibitor enhanced the ability of colony formation in GC cells (Fig. 3h–i). Consistently, these phenomena were rigorously explained in the immunoblotting assays, as indicated in Fig. 3j–k, tRF-Val-CAC-016 mimics obviously declined the protein expression of CyclinD1, CyclinB, c-myc. tRF-Val-CAC-016 inhibitor increased the protein expression of CyclinD1, CyclinB, c-myc. On the other hand, oxaliplatin decreased the expression of CyclinD1, c-myc compared with the control.

### Bioinformatics analysis

**Fig. 4:**
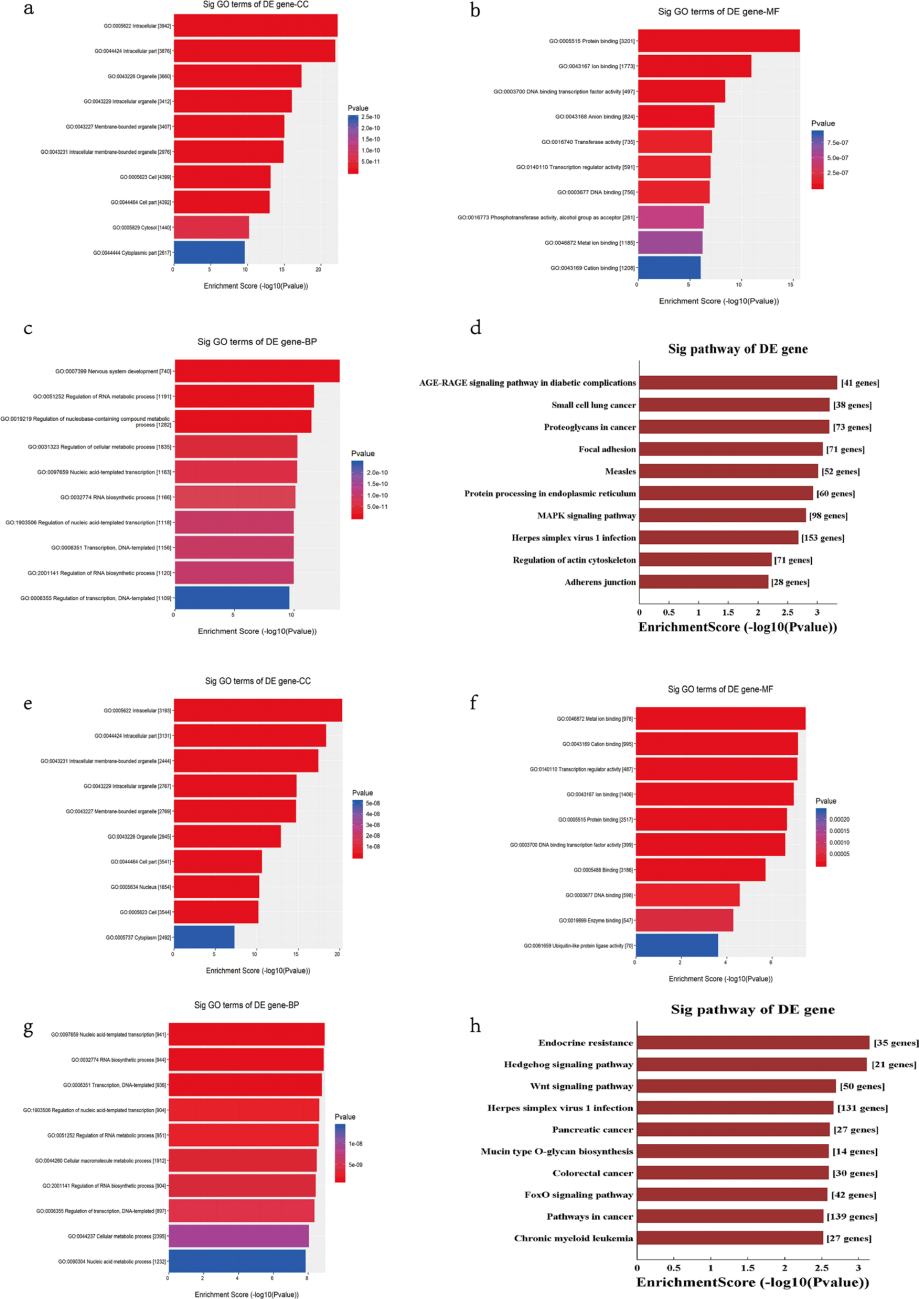
GO and KEGG enrichment analysis of sequencing profiles. **a**–**d** GO and KEGG enrichment analysis for the target genes of six down-regulated tsRNAs (tRF-Val-CAC-016, tRF-Glu-TTC-027, tRF-Glu-TTC-026, tRF-Ser-TGA-011, tiRNA-Pro-TGG-001, tRF-Ser-GCT-113). **e–h** GO and KEGG enrichment analysis for the four up-regulated tsRNAs (tiRNA-Val-CAC-001, tiRNA-His-GTG-001, tRF-Glu-TTC-017, tiRNA-Asp-GTC-001)

We applied miRanda and TargetScan databases to predict the target genes of tsRNAs. Based on the matching of mRNA and tsRNAs expression profile data, some biological site-sequence features of significance and scoring models with relative conservation were uncovered. Parameters such as structure score, energy and context_plus_score were used to optimize the selection of the target genes of tsRNAs [21, 22]. Then target genes of the down-regulated or the up-regulated tsRNAs were then enriched in the GO and KEGG analysis. In the down-regulated group, GO analysis was presented in Fig. 4a–c, and we found that the MAPK signaling pathways were enriched significantly (Fig. 4d). In the up-regulated group, GO analysis was shown in Fig. 4e–g, and we found that the Wnt signaling pathway was quite prominent (Fig. 4h). We then compared the bioinformatics data in the present study with GEO (Additional file 4: Fig. S2) and TCGA databases (Additional file 5: Fig. S3), which suggested that proliferation-related pathways were frequently enriched (Additional file 4: Fig. S2f and Additional file 5: Fig. S3f), and the Calcium signaling pathway was uncovered (Additional file 5: Fig. S3i), consistent with the function of *CACNA1d.*

### *CACNA1d* was verified up-regulated in GC tissues and was selected as the potential target of tRF-Val-CAC-016

**Fig. 5:**
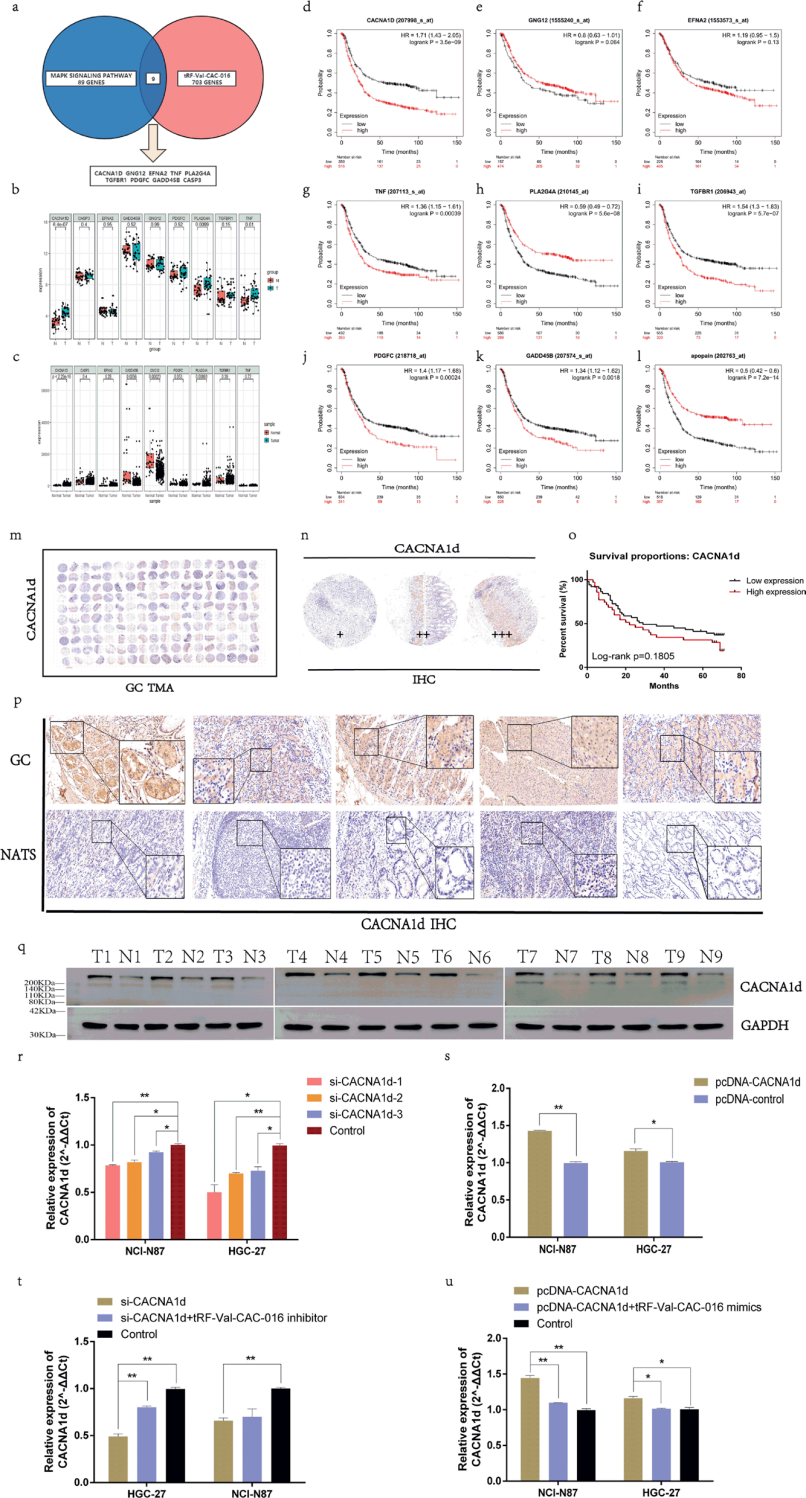
CACNA1d was verified up-regulated in GC tissues and was selected as the potential target of tRF-Val-CAC-016. **a** The Venn diagram took the overlap of MAPK components and the predicted target genes of tRF-Val-CAC-016. **b–c** *CACNA1d*, *PLA2G4A,* and *TNF* in GSE65801, *CACNA1d,* and *PLA2G4A* in TCGA-STAD were significantly up-regulated. **d–l** *CACNA1d*, *TNF*, *TGFBR1*, *PDGFC*, *GADD45B* were significantly and negatively related to the prognosis of GC. **m–n** The tissue microarray (TMA) with 90 pairs of GC specimens, including detailed follow-up data. **o** The expression of *CACNA1d* was not significantly related to the prognosis of GC (*p* = 0.1805). **p** Ten representative IHC images of TMA were presented, including GC and NATS. **q** The protein levels of *CACNA1d* were highly expressed in nine GC tissues compared with corresponding NATs. **r** si-*CACNA1d*-1 was a better inhibitor for *CACNA1d* compared with si-*CACNA1d*-2 and si-*CACNA1d*-3. **s** pcDNA-*CACNA1d* could significantly enhance the expression of *CACNA1d*. **t** The tRF-Val-CAC-016 inhibitor was able to reverse the suppressive function of si-*CACNA1d* on GC cells to some extent. **u** The effect of pcDNA-*CACNA1d* on GC was partly relieved by tRF-Val-CAC-016 mimics. *P < 0.05, **P < 0.01, statistically significant

We introduced the TCGA and GEO databases to predict the possible target to conduct further research. As shown in Fig. 5a, the Venn diagram took the overlap of MAPK components and the predicted target genes of tRF-Val-CAC-016. Then we analyzed the GEO database (GSE65801) and TCGA-STAD database and found that *CACNA1d*, *PLA2G4A* and *TNF* in GSE65801 (Fig. 5b), *CACNA1d* and *PLA2G4A* in TCGA-STAD were significantly up-regulated (Fig. 5c). Furthermore, we then applied the Kaplan–Meier plotter website and discovered that *CACNA1d*, *TNF*, *TGFBR1*, *PDGFC*, *GADD45B* were significantly and oppositely related to the prognosis of GC (Fig. 5d–l). Analysis above reminded us the vital role of *CACNA1d* as the possible downstream target of tRF-Val-CAC-016. Subsequently, we obtained the tissue microarray (TMA) with 90 pairs of GC specimens, including detailed follow-up data (Fig. 5m). IHC results were presented in Fig. 5n. Through the analysis of the follow-up data, we found that the expression of *CACNA1d* was not significantly related to the prognosis of GC (*p* = 0.1805) (Fig. 5o). The representative IHC images of GC and NATs are shown in Fig. 5p. However, the protein levels of *CACNA1d* in GC tissues were up-regulated compared with corresponding NATs (Fig. 5q). Hence, we selected *CACNA1d* as the target gene based on the intersection of the comprehensive analysis. To confirm the expression and function of *CACNA1d,* we purchased siRNAs for *CACNA1d* and selected si-*CACNA1d*-1 as the better inhibitory effect compared with si-*CACNA1d*-2 and si-*CACNA1d*-3 (Fig. 5r). On the contrary, pcDNA-*CACNA1d* could significantly enhance the expression of *CACNA1d* (Fig. 5s)*.* Moreover, the tRF-Val-CAC-016 inhibitor was able to reverse the suppressive function of si-*CACNA1d* on GC cells to some extent (Fig. 5t). Analogously, the effect of pcDNA-*CACNA1d* on GC was partly relieved by tRF-Val-CAC-016 mimics (Fig. 5u). The sequences of *CACNA1d* primers and siRNAs are listed in Additional file 3: Table S2.

### tRF-Val-CAC-016 was immunoprecipitated by Argonaute-2 and could modulate the proliferation of GC by targeting *CACNA1d*

**Fig. 6:**
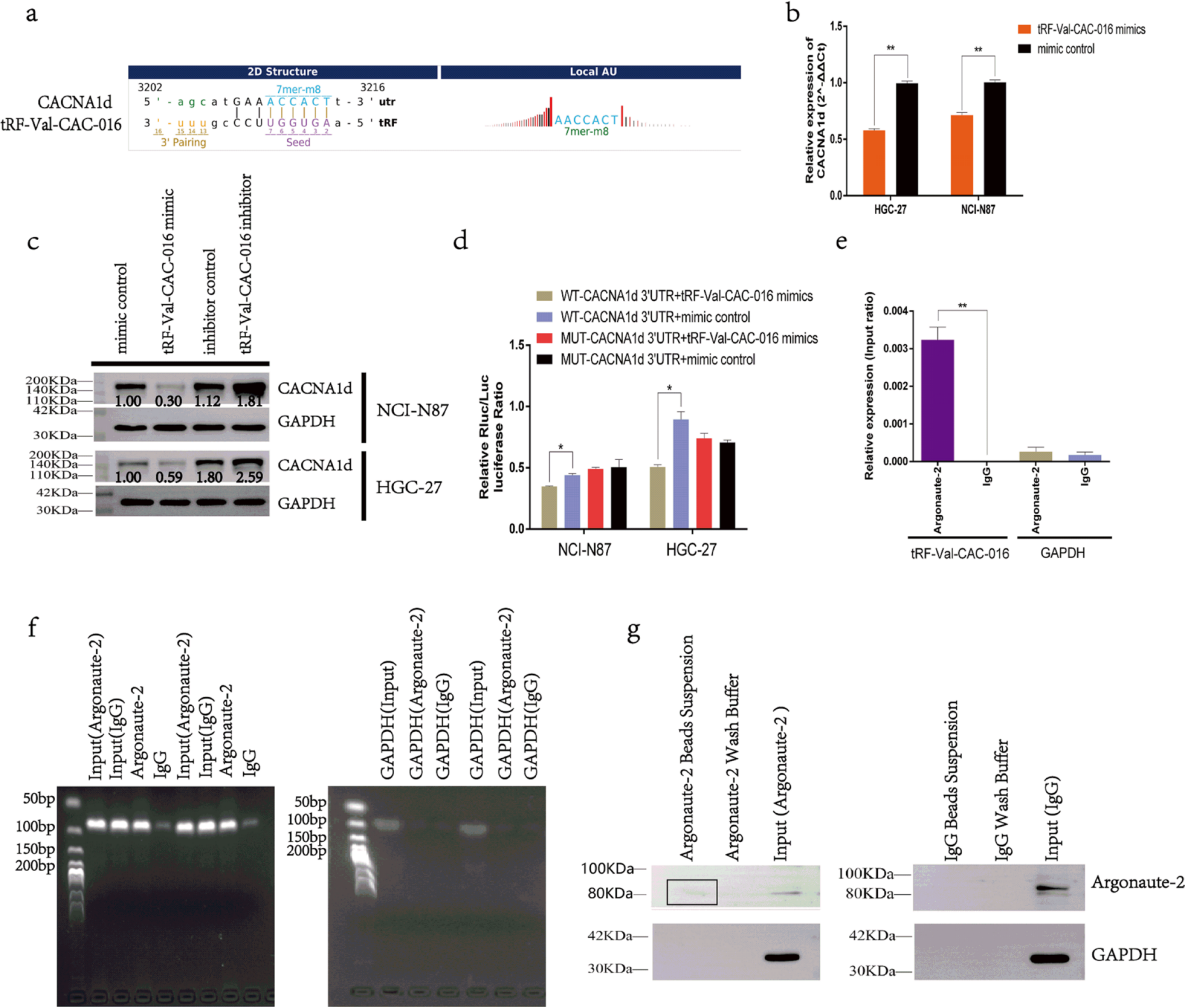
tRF-Val-CAC-016 was immunoprecipitated by Argonaute-2 and could modulate the proliferation of GC by targeting *CACNA1d*. **a** Binding relation between tRF-Val-CAC-016 and *CACNA1d* mRNA. **b–c** tRF-Val-CAC-016 mimics could significantly reduce the expression of *CACNA1d* using RT-PCR and immunoblotting*.* **d** Dual-luciferase reporter assay indicated that WT-*CACNA1d*-3′UTR plus tRF-Val-CAC-016 mimics group could significantly reduce the luciferase ratio compared with other groups in NCI-N87 and HGC-27. **e** In RIP assay, tRF-Val-CAC-016 was significantly immunoprecipitated by Argonaute-2 compared with the IgG group. **f–g** Gel-electrophoresis further verified the PCR product of RIP assays, and the immunoblotting confirmed the integrity of the process to wash the magnetic beads. *P < 0.05, **P < 0.01, statistically significant

The sequencing profile has elucidated the possible binding relation between tRF-Val-CAC-016 and *CACNA1d* mRNA (Fig. 6a), and the tRF-Val-CAC-016 mimics could significantly reduce the expression of *CACNA1d* in RT-PCR (Fig. 6b)*.* The protein level of *CACNA1d* was decreased by tRF-Val-CAC-016 mimics and promoted by tRF-Val-CAC-016 inhibitor (Fig. 6c). Subsequently, we found that WT-*CACNA1d*-3′UTR plus tRF-Val-CAC-016 mimics group could significantly reduce the luciferase ratio compared with other groups in the Dual-luciferase reporter assay (Fig. 6d). Then we introduced RIP (RNA-binding protein immunoprecipitation) assay and found that tRF-Val-CAC-016 was significantly immunoprecipitated by Argonaute-2 compared with the IgG group (Fig. 6e). Gel-electrophoresis further verified the PCR product of RIP assays (Fig. 6f). Furthermore, the immunoblotting confirmed the integrity of the process to wash the magnetic beads (Fig. 6g).

### *CACNA1d* strengthened the proliferation of GC and was modulated by tRF-Val-CAC-016

**Fig. 7:**
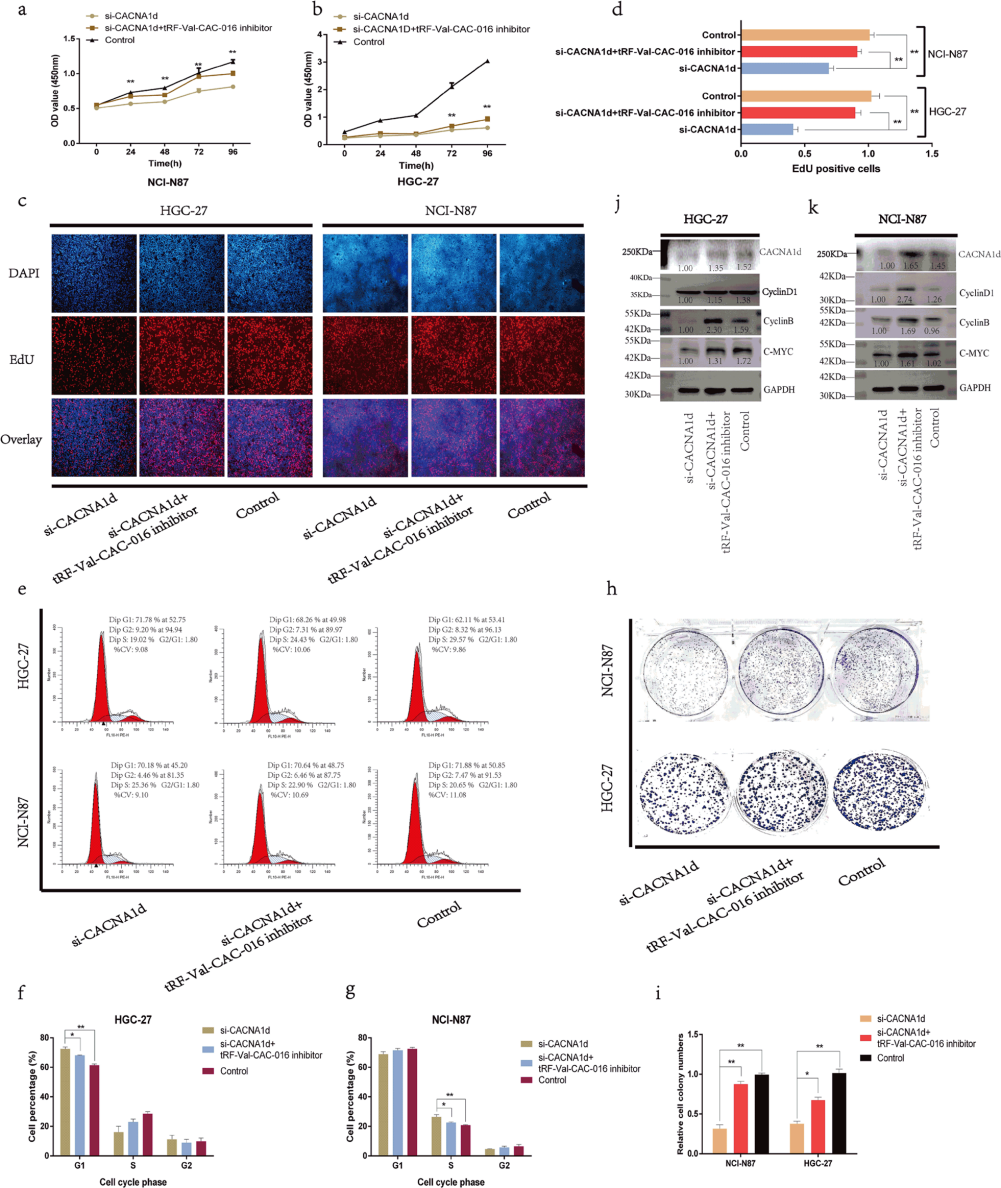
si-*CACNA1d* inhibited the proliferation of GC and could be rescued by tRF-Val-CAC-016 inhibitor. **a**–**b** In the CCK-8 assays, si-*CACNA1d* could significantly decline the proliferation of GC cells and was partly recovered by tRF-Val-CAC-016 inhibitor in HGC-27 and NCI-N87. **c–d** In the EdU assays, the tRF-Val-CAC-016 inhibitor could rescue the inhibitory effect of si-*CACNA1d* in terms of cell replication activity. **e–g** si-*CACNA1d* resulted in the G1 phase arrest in HGC-27, but S phase arrest in NCI-N87 with flow cytometry and could both be rescued by tRF-Val-CAC-016 inhibitor. **h–i** The rescue effect of the tRF-Val-CAC-016 inhibitor on si-*CACNA1d* was also confirmed in the colony formation assays. **j–k** The suppressive function of si-*CACNA1d* on the protein expression of *CACNA1d,* CyclinD1, CyclinB, c-myc was partly resumed by tRF-Val-CAC-016 inhibitor. *P < 0.05, **P < 0.01, statistically significant

**Fig. 8:**
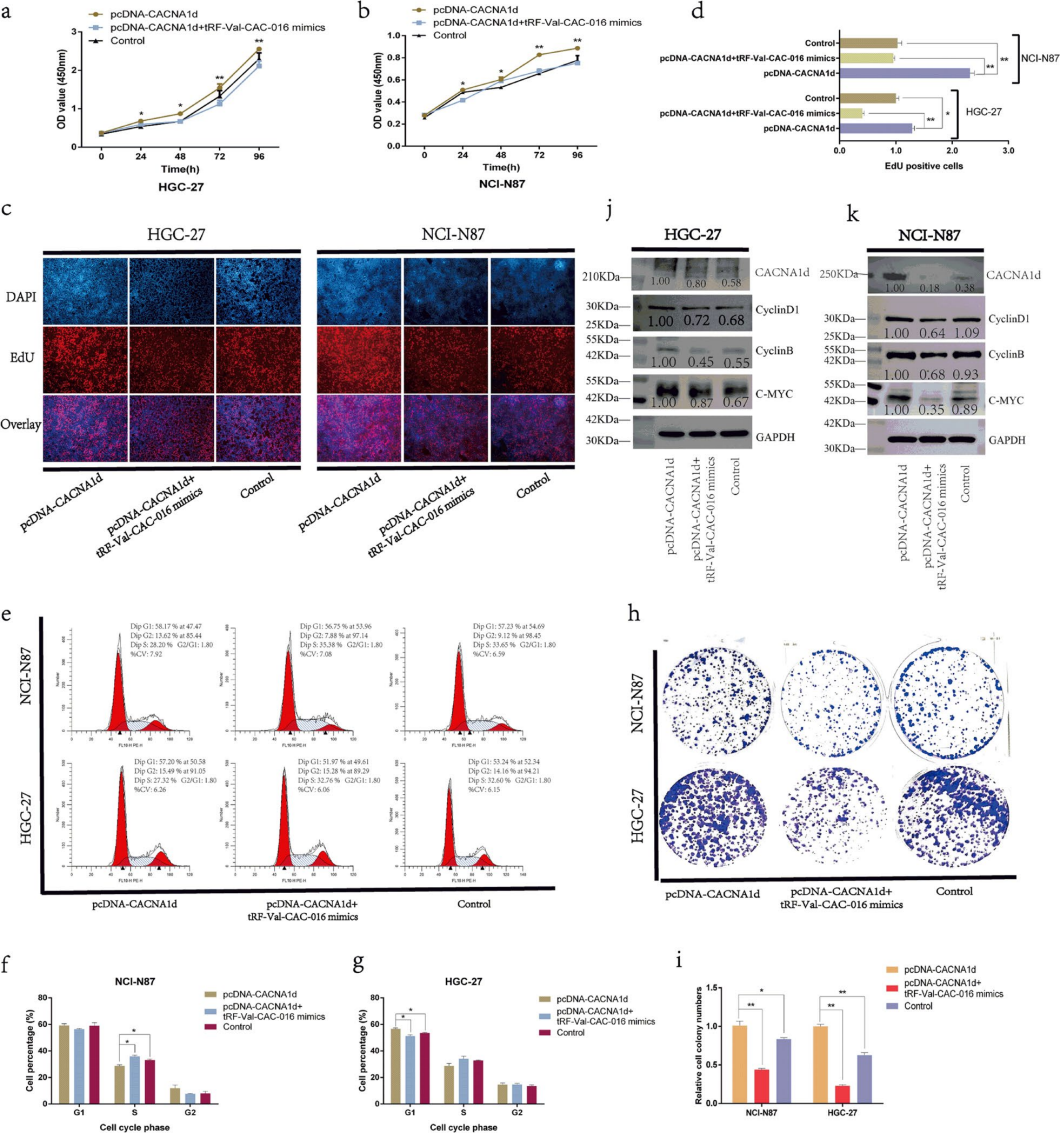
pcDNA-*CACNA1d* strengthened the proliferation of GC and was modulated by tRF-Val-CAC-016 mimics. tRF-Val-CAC-016 mimics could significantly reverse the reinforced effect of pcDNA-*CACNA1d* on the proliferation of GC cells in terms of the CCK-8 (**a–b**), EdU (**c–d**), flow cytometry for cell cycle (**e–g**) and colony formation assays (**h–i**). **j–k** pcDNA-*CACNA1d* significantly lifted the protein levels of *CACNA1d,* CyclinD1, CyclinB, c-myc, and tRF-Val-CAC-016 mimics could decline this function. *P < 0.05, **P < 0.01, statistically significant

Rescue assays were undertaken to elucidate the connection between tRF-Val-CAC-016 and *CACNA1d* further*.* In the CCK-8 assays, si-*CACNA1d* could significantly decline the proliferation of GC cells, which was partially recovered by tRF-Val-CAC-016 inhibitor (Fig. 7a–b). In the EdU assays, the tRF-Val-CAC-016 inhibitor could rescue the inhibitory effect of si-*CACNA1d* in terms of cell replication activity (Fig. 7c–d). Interestingly, si-*CACNA1d* resulted in the G1 phase arrest in HGC-27 but S phase arrest in NCI-N87, and could both be rescued by tRF-Val-CAC-016 inhibitor (Fig. 7e–g). Similarly, the rescue effect of the tRF-Val-CAC-016 inhibitor on si-*CACNA1d* was also confirmed in the colony formation assays (Fig. 7h–i). The suppressive function of si-*CACNA1d* on the protein expression of *CACNA1d,* CyclinD1, CyclinB, c-myc was partly resumed by tRF-Val-CAC-016 inhibitor (Fig. 7j–k). Analogously, the reverse verification for the function of *CACNA1d* was then performed. pcDNA-*CACNA1d* and pcDNA-*CACNA1d* plus tRF-Val-CAC-016 mimics were transfected, respectively. As shown in Fig. 8a–i, tRF-Val-CAC-016 mimics could significantly reverse the reinforced effect of pcDNA-*CACNA1d* on the proliferation of GC cells. Moreover, pcDNA-*CACNA1d* significantly lifted the protein levels of *CACNA1d,* CyclinD1, CyclinB, c-myc, but tRF-Val-CAC-016 mimics could decline this function (Fig. 8j–k).

### Inhibitor of MAPK signaling pathway (p38 MAPK-IN) significantly reversed the enhancement of tRF-Val-CAC-016 inhibitor on GC proliferation

**Fig. 9:**
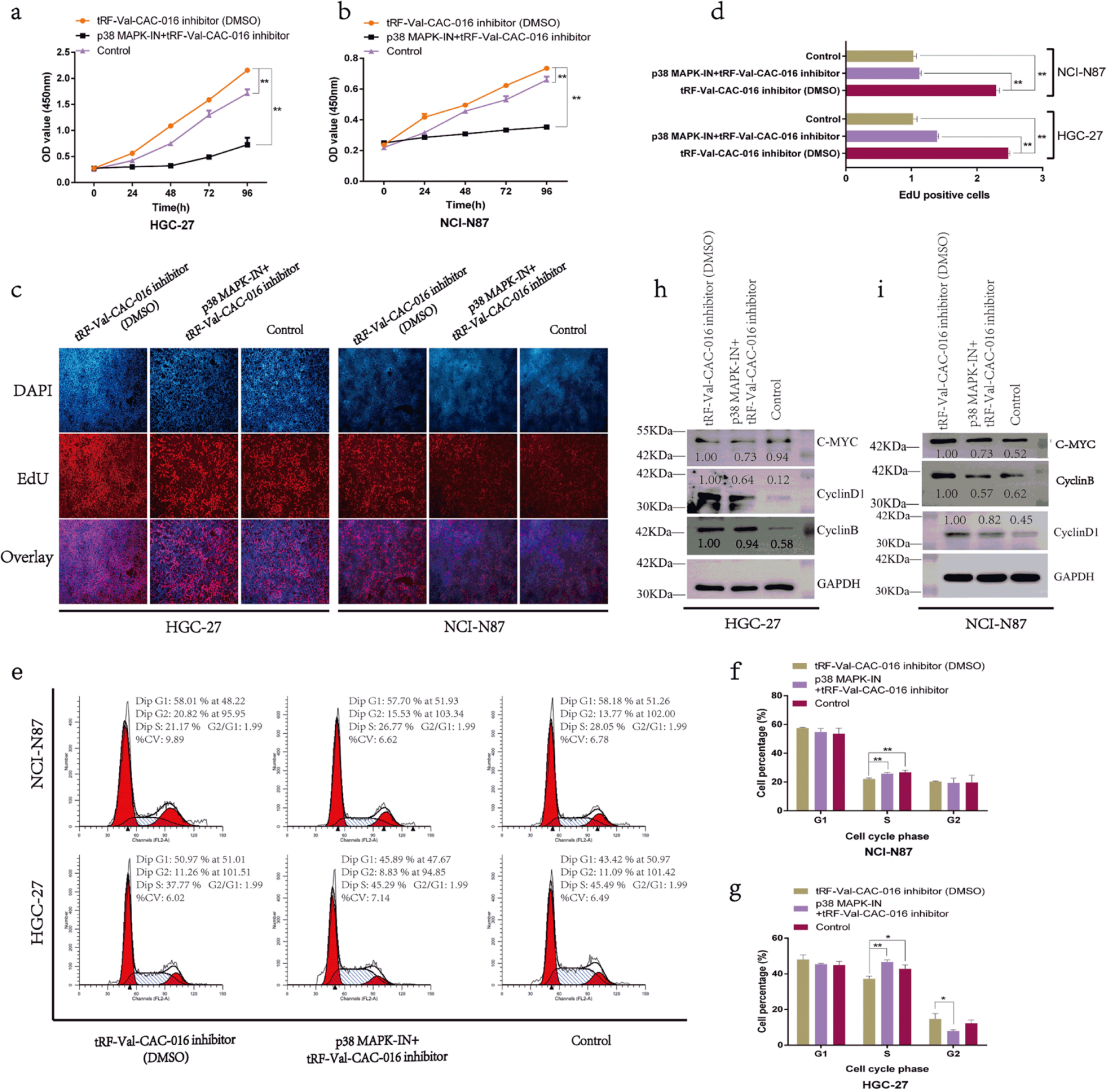
Inhibitor of MAPK signaling pathway (p38 MAPK-IN) significantly reversed the enhancement of tRF-Val-CAC-016 inhibitor on GC proliferation. **a**–**b** tRF-Val-CAC-016 inhibitor enhanced the GC proliferation in the CCK-8 assay, which p38 MAPK-IN could partly reverse. **c–d** tRF-Val-CAC-016 inhibitor promoted the cell replication activity, and p38 MAPK-IN could decline this activity in HGC-27 and NCI-N87. The reversal phenomena also happened in the flow cytometry assays for cell cycle (**e–g**) and immunoblotting assays for *CACNA1d,* CyclinD1, CyclinB, c-myc (**h–i**). *P < 0.05, **P < 0.01, statistically significant

To demonstrate the role of the MAPK signaling pathway, we took p38 MAPK-IN to rescue the enhancement of tRF-Val-CAC-016 inhibitor on GC proliferation. It indicated that p38 MAPK-IN was able to block the pathway conduction partly as expected, which was manifested in the aspect of the deterioration to the influence of tRF-Val-CAC-016 inhibitor on GC. As shown in Fig. 9a–b, tRF-Val-CAC-016 inhibitor enhanced the GC proliferation in the CCK-8 assay, which p38 MAPK-IN could partly reverse. Meanwhile, tRF-Val-CAC-016 inhibitor promoted the cell replication activity, and p38 MAPK-IN could also decline this activity (Fig. 9c–d). The reversal phenomena also happened in the flow cytometry assays for cell cycle (Fig. 9e–g), and immunoblotting assays for *CACNA1d,* CyclinD1, CyclinB, c-myc (Fig. 9h–i).

### tRF-Val-CAC-016 suppressed tumor proliferation in NCI-N87 xenografts

**Fig. 10:**
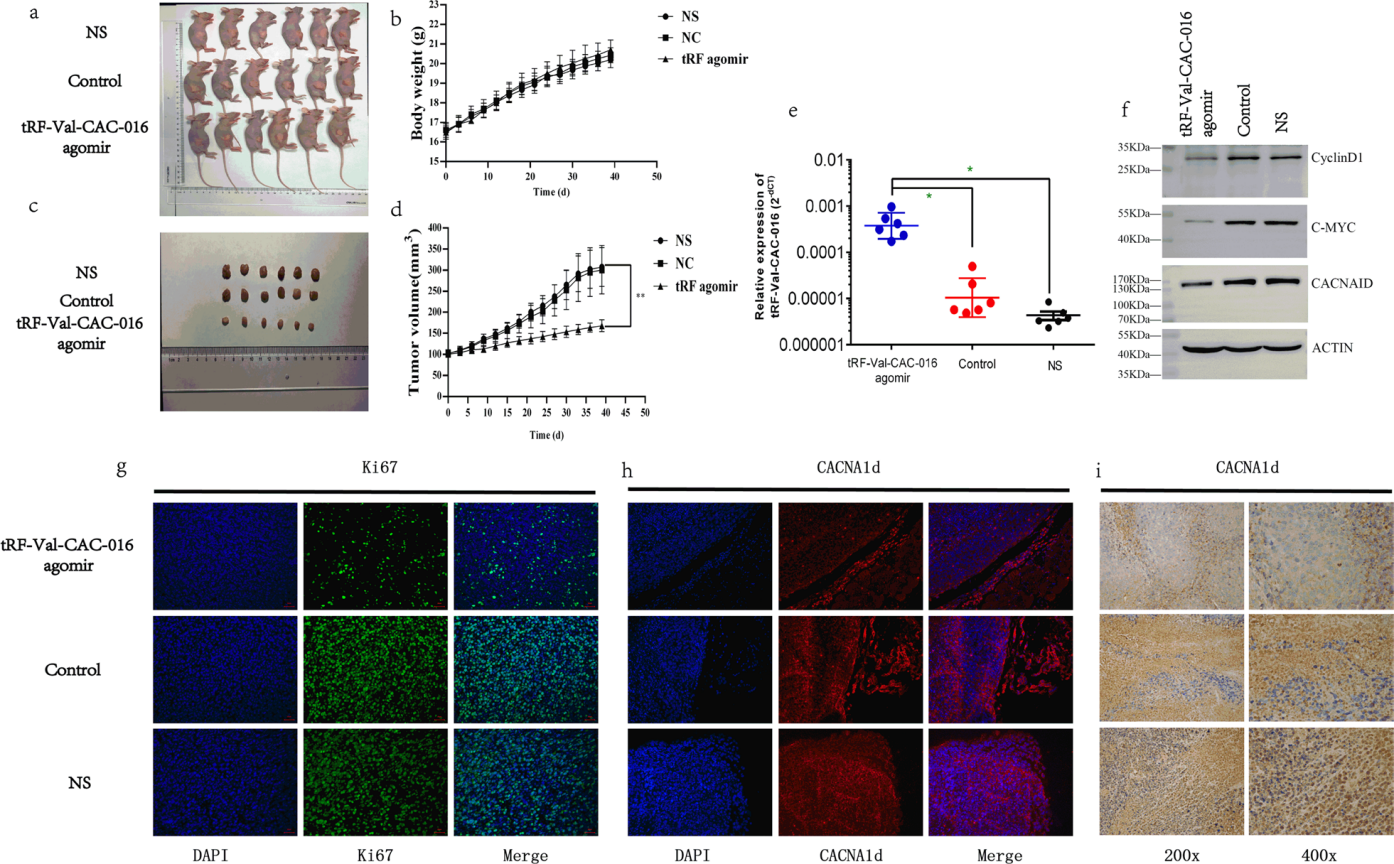
tRF-Val-CAC-016 inhibited tumor proliferation in the NCI-N87 xenografts. **a** Representative images of NCI-N87 xenografts in vivo. Six mice are included in each group. **b–d** The results indicated that tRF-Val-CAC-016 declined tumor growth capacity in mice in the aspect of the bodyweight of the xenografts and the tumor volumes. **e** The PCR results of these resected tumors showed that tRF-Val-CAC-016 agomir significantly increased the expression of tRF-Val-CAC-016 compared with the agomir control group and normal saline (NS) group. **f** The immunoblotting assays showed the inhibitory effect of tRF-Val-CAC-016 agomir on CACNA1d, c-myc, and cyclinD1. **g–h** The immunofluorescence staining assays for *ki-67* and *CACNA1d.* **i** The Immunohistochemistry (IHC) assays demonstrated the low expression of *CACNA1d* in the tRF-Val-CAC-016 agomir group compared with the agomir control group and normal saline (NS) group. *P < 0.05, **P < 0.01, statistically significant

Subcutaneous xenograft experiments were undertaken to discuss the function of tRF-Val-CAC-016 in vivo*.* The results indicated that tRF-Val-CAC-016 declined the capacity of tumor growth in mice in the aspect of the bodyweight of the xenografts and the tumor volumes (Fig. 10a–d). The PCR results of these resected tumors showed that tRF-Val-CAC-016 agomir significantly suppressed the proliferation compared with the agomir control group and normal saline (NS) group (Fig. 10e), which also happened in the immunoblotting assays for these mice tumors (Fig. 10f). Besides, the immunofluorescence staining assays for *ki-67* and *CACNA1d* suggested that tRF-Val-CAC-016 reduced the proliferative capacity of GC and further confirmed the expression and location of *CACNA1d* protein (Fig. 10g–h)*.* Meanwhile, the Immunohistochemistry (IHC) assays also demonstrated the low expression of *CACNA1d* in the tRF-Val-CAC-016 agomir group (Fig. 10i).

### tRNA derivative tRF-Val-CAC-016 modulates the canonical MAPK signaling pathways by targeting *CACNA1d*

**Fig. 11:**
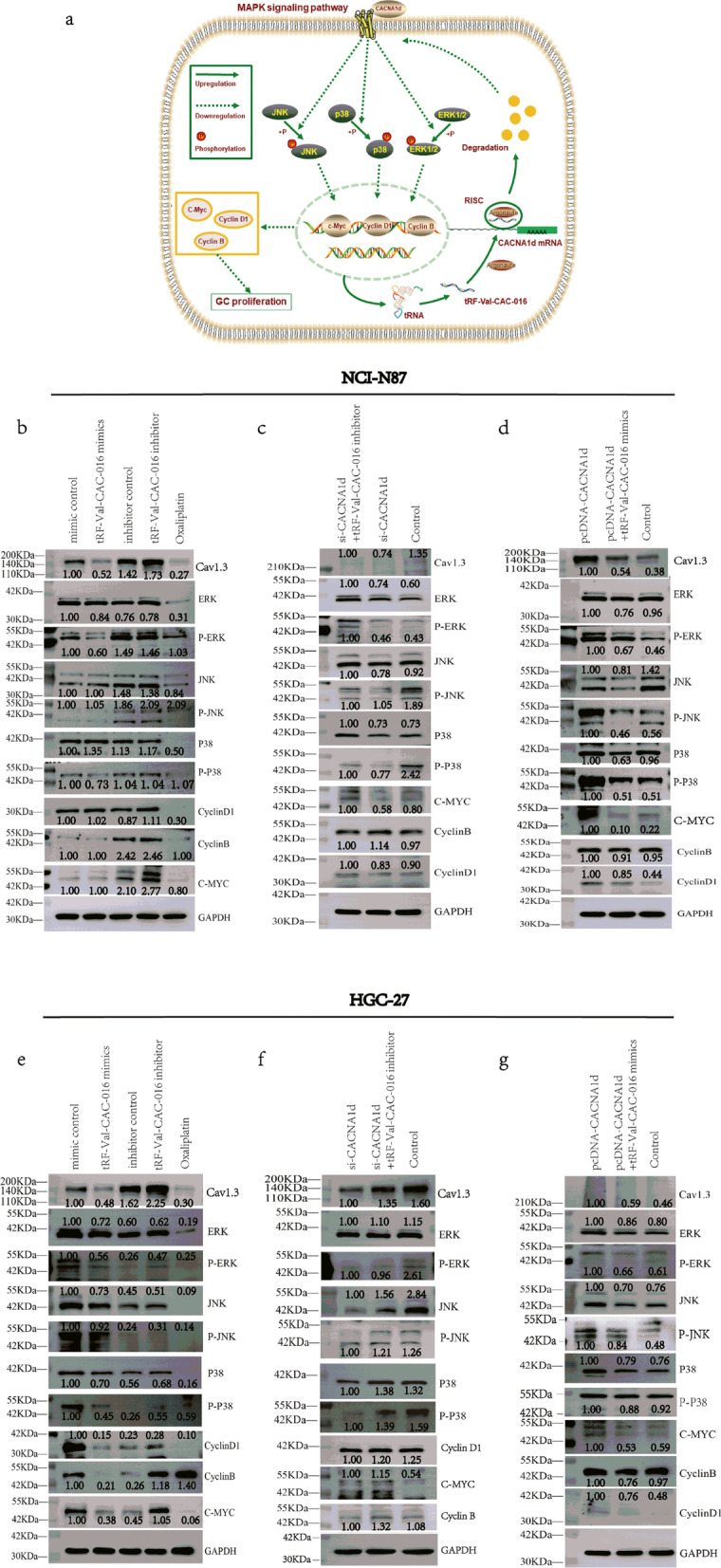
tRNA derivative tRF-Val-CAC-016 modulates the canonical MAPK signaling pathway by targeting *CACNA1d*. **a** The mechanism diagram clearly explained the pattern that tRF-Val-CAC-016 exerted its influence on the MAPK signaling pathway. **b** In the immunoblotting assays of NCI-N87, tRF-Val-CAC-016 mimics suppressed the expression of *CACNA1d* (*Cav1.3*), ERK, p-ERK, and p-p38. tRF-Val-CAC-016 inhibitor enhanced the expression of *CACNA1d* (*Cav1.3*), ERK, p-JNK, p38, cyclinB, cyclinD1, c-myc. **c** si-*CACNA1d* could inhibit the expression levels of *CACNA1d* (*Cav1.3*), JNK, p-p38, cyclinD1, and c-myc, which was able to be reversed by tRF-Val-CAC-016 inhibitor. **d** pcDNA-*CACNA1d* increased the expression of *CACNA1d* (*Cav1.3*), ERK, p-ERK, p-JNK, p38, p-p38, cyclinB, cyclinD1, and c-myc, which was rescued by tRF-Val-CAC-016 mimics. **e** In HGC-27, tRF-Val-CAC-016 mimics declined, but tRF-Val-CAC-016 inhibitor promoted the protein levels of *CACNA1d* (*Cav1.3*), ERK, p-ERK, JNK, p-JNK, p38, p-p38, cyclinB, cyclinD1, c-myc. **f** *CACNA1d* (*Cav1.3*), ERK, JNK, p-JNK, p38, p-p38, cyclinB, and cyclinD1 were down-regulated by si-*CACNA1d*, and this effect was reversed by tRF-Val-CAC-016 inhibitor. **g** *CACNA1d* (*Cav1.3*), ERK, p-ERK, JNK, p-JNK, p38, p-p38, cyclinB, cyclinD1, and c-myc were enhanced by pcDNA-*CACNA1d*, which could be deteriorated by tRF-Val-CAC-016 mimics

As presented in Fig. 11a, the mechanism diagram clearly explained the pattern that tRF-Val-CAC-016 exerted its influence on the MAPK signaling pathway. Overview of MAPK signaling pathway was shown in Additional file 6: Fig. S4. In the immunoblotting assays of NCI-N87, tRF-Val-CAC-016 mimics suppressed the expression of *CACNA1d* (*Cav1.3*), ERK, p-ERK, and p-p38. tRF-Val-CAC-016 inhibitor enhanced the expression of *CACNA1d* (*Cav1.3*), ERK, p-JNK, p38, cyclinB, cyclinD1, c-myc (Fig. 11b). si-*CACNA1d* could inhibit the expression levels of *CACNA1d* (*Cav1.3*), JNK, p-p38, cyclinD1, and c-myc, which was able to be reversed by tRF-Val-CAC-016 inhibitor (Fig. 11c). pcDNA-*CACNA1d* increased the expression of *CACNA1d* (*Cav1.3*), ERK, p-ERK, p-JNK, p38, p-p38, cyclinB, cyclinD1, and c-myc, which was rescued by tRF-Val-CAC-016 mimics (Fig. 11d). In HGC-27, tRF-Val-CAC-016 mimics declined, but tRF-Val-CAC-016 inhibitor promoted the protein levels of *CACNA1d* (*Cav1.3*), ERK, p-ERK, JNK, p-JNK, p38, p-p38, cyclinB, cyclinD1, c-myc (Fig. 11e). *CACNA1d* (*Cav1.3*), ERK, JNK, p-JNK, p38, p-p38, cyclinB, and cyclinD1 were down-regulated by si-*CACNA1d*, and this effect was reversed by tRF-Val-CAC-016 inhibitor (Fig. 11f). *CACNA1d* (*Cav1.3*), ERK, p-ERK, JNK, p-JNK, p38, p-p38, cyclinB, cyclinD1, and c-myc were enhanced by pcDNA-*CACNA1d*, which could be deteriorated by tRF-Val-CAC-016 mimics (Fig. 11g).

## Discussion

tsRNAs are a class of ubiquitously expressed and conserved ncRNAs in nature. Emerging studies suggest that tsRNAs have been a rising star in this field of cancer research [7, 23–30].

The results of a considerable amount of studies were consistent with our present study regarding the role of tsRNAs as a regulator in the aspect of tumor progression [7, 31–39]. Farina et al. found that RUNX1 could maintain the mammary epithelium by repressing ts-112 [40]. Kim et al. discovered that LeuCAG3′tsRNA might maintain ribosomal protein S28 (RPS28) levels after translation initiation to explain why some mRNA levels do not necessarily correlate with protein levels [41]. Wang et al. identified the novel tsRNA-06018 as a critical regulator in hMSC adipogenesis [42]. Generally speaking, a considerable amount of studies mainly focused on the post-transcriptional regulation to influence the translation of specific proteins. However, many researchers also found the critical function of tsRNAs as the biomarkers in diagnosing diseases [5, 43, 44]. Meanwhile, many tsRNAs were found related to several signaling pathways to facilitate the modulation of certain tumors’ progression. For instance, Wang et al. reported that tiRNA^Tyr−GTA^ could modulate the peroxisome proliferator-activated receptor signaling pathway [45]. Furthermore, tRF/miR-1280 was discovered to maintain the function of cancer stem cell-like cells of CRC by inhibiting the Notch signaling pathway [26]. Balatti et al. applied CRISPR technology to generate ts-101 and ts-46 KO stable cell lines from HEK293 cells, then Affymetrix gene-expression profiling was performed to screen the differentially expressed genes. Finally, they discovered that tsRNAs could regulate the chromatin configuration and the epigenetic control of gene expression. In addition, several signaling pathways related to cell transformation and cancer development, such as PDGF signaling, mTOR signaling, and PTEN signaling, were also found to be modulated [46]. Our study demonstrated the critical role of tRF-Val-CAC-016 in the activation of the MAPK signaling pathway, which was also noticeable in the field of tsRNAs research.

In terms of the present research, we selected tRF-Val-CAC-016 as the research target. Interestingly, tRF-Val-CAC-016 was significantly low-expressed in GC cell lines (NCI-N87 and HGC-27) and tissues compared with GES-1 and NATs, respectively. However, the expression levels of GC and NATs were not grouped consistently as the heterogeneity of tumors. The expression of tumors may be closely related to the differentiation, histology, location, and even sampling methods. Besides, individual differences also need to be fully recognized. Therefore, a slight inconsistency in expression levels is acceptable. Subsequently, by the overexpression of tRF-Val-CAC-016, we observed that tRF-Val-CAC-016 could suppress the proliferation of GC cells. Through the RIP assay and Dual-luciferase reporter assay, we rigorously confirmed the bioinformatic results concerning the binding relation between tRF-Val-CAC-016 and *CACNA1d.* As *CACNA1d* is a member of the MAPK signaling pathway, we assumed that tRF-Val-CAC-016 might modulate the GC proliferation via tRF-Val-CAC-016/ *CACNA1d/* MAPK signaling pathways axis. Through the rescue assays, we confirmed the regulative relationships among tRF-Val-CAC-016, *CACNA1d,* and MAPK signaling pathway. We conducted the subcutaneous xenograft experiments to verify the function of tRF-Val-CAC-016 in vivo further*.* Subsequently, the results of immunoblotting assays related to the MAPK signaling pathway were consistent with our hypothesis that tRF-Val-CAC-016 modulates the canonical MAPK signaling pathways by targeting *CACNA1d* to influence the proliferation of gastric carcinoma. Hence, these findings uncovered a remarkable role of tRF-Val-CAC-016 as a tumor inhibitor in GC.

This study preliminarily explored the mechanism that tRF-Val-CAC-016 participated in to regulate the proliferation of GC. However, there still exist a few deficiencies in this research. For instance, the detailed mechanism of tRNAs splicing to produce tsRNAs was not investigated by us thoroughly and the regulatory network of the significant tsRNAs is not analyzed comprehensively. The crosstalking among the tsRNAs needs to be discovered in further research.

## Conclusions

In summary, this research illustrated the function of tRF-Val-CAC-016 in GC for the first time. The discussion for the mechanism tRF-Val-CAC-016 was involved in was relatively comprehensive to some extent. This study showed that tRF-Val-CAC-016 was down-regulated significantly in GC, and the expression was related to the histology and tumor size in terms of the clinicopathological analysis. Besides, tRF-Val-CAC-016 modulates the transduction of *CACNA1d*-mediated MAPK signaling pathways to suppress the proliferation of gastric carcinoma. Taken together, tRF-Val-CAC-016 could be a potential diagnostic biomarker and a new therapeutic target for GC.

## Supplementary Information

**Additional file 1.Table S1**. Information of antibodies for Western blot, immunofluorescence staining and IHC.

**Additional file 2: Figure S1.** Sequencing profiles of tsRNAs. **a**–**b** Pie charts of the distribution of tsRNA subtypes. The values in the bracket represent the number of tsRNA subtypes. The colors represent the subtypes of tsRNA. **c–d** The frequencies of tsRNA subtypes with different lengths. The X-axis represents the length of tsRNA, and the Y-axis shows the frequency of tsRNA. The colors represent distinct subtypes of tsRNA. **e–f** The number of different kinds of tsRNA isodecoders in GC tissues. The X-axis represents the types of tsRNA isodecoders, and the Y-axis shows the number of tsRNA isodecoders. The colors represent different subtypes of tsRNA.

**Additional file 3.Table S2**. The sequence of siRNAs and tRF mimics.

**Additional file 4: Figure S2.** Bioinformatics results of GEO database. **a** Heatmap for hierarchical clustering; **b** Volcano plots; **c–e** Gene Ontology (GO) analysis; **f** KEGG analysis; **g–h** Gene Set Enrichment Analysis (GSEA).

**Additional file 5: Figure S3.** Bioinformatics results of TCGA-STAD database. **a** Heatmap for hierarchical clustering; **b** Volcano plots; **c–e** Gene Ontology (GO) analysis; **f** KEGG analysis; **g–i** Gene Set Enrichment Analysis (GSEA).

**Additional file 6: Fig. S4.** Overview of the MAPK signaling pathway.

## Data Availability

The datasets used and/or analysed during the current study are available from the corresponding author on reasonable request.

## Abbreviations

tRFs: TRNA-derived fragments
tiRNAs: TRNA halves
GC: Gastric Cancer
TNM: Tumor-Node-Metastasis
RT-PCR: Real time-PCR
GSEA: Gene Set Enrichment Analysis
GO: Gene Ontology
KEGG: Kyoto Encyclopedia of Genes and Genomes
NATs: Non-tumor Adjacent Tissues
DETs: Differentially Expressed tsRNAs
NDETs: Not Differentially Expressed tsRNAs
RIP: RNA-binding protein immunoprecipitation
EdU: Ethynyl-2′-deoxyuridine
FISH: Fluorescent In Situ Hybridization
RT: Room Temperature
NS: Normal Saline
FC: Fold-Change
