# Supplementary material for: tRF-Val-CAC-016 modulates the transduction of CACNA1d-mediated MAPK signaling pathways to suppress the proliferation of gastric carcinoma
Source: Cell Commun Signal. 2022 May 19;20:68. doi: 10.1186/s12964-022-00857-9 (PMC9118711; doi:10.1186/s12964-022-00857-9)
Supplement: Supplementary file 2 — Additional file 1.Table S1. Information of antibodies for Western blot, immunofluorescence staining and IHC. [file 12964_2022_857_MOESM2_ESM.docx]

**Table S1 Information of antibodies for Western blot, immunofluorescence staining and IHC.**

| **Antibody** | **Manufacturer** | **Item NO.** | **Species** | **Dilution (primary)** |
| --- | --- | --- | --- | --- |
| CACNA1d | abcam | Ab84811 | Mouse | 1: 1000 |
| ERK1/2 | Servicebio | GB11560 | Rabbit | 1: 1000 |
| p-ERK1/2 | CST | 4370 | Rabbit | 1: 1000 |
| JNK | proteintech | 24164-1-AP | Rabbit | 1: 1000 |
| p-JNK | CST | 4668 | Rabbit | 1: 1000 |
| p38 | abcam | ab32142 | Rabbit | 1: 1000 |
| p-p38 | CST | 4511 | Rabbit | 1: 1000 |
| c-Myc | proteintech | 10828-1-AP | Rabbit | 1: 1000 |
| CyclinB | abcam | ab185622 | Rabbit | 1: 1000 |
| CyclinD1 | proteintech | 60186-1-LG | Mouse | 1: 1000 |
| Ki67 | abcam | ab16667 | Rabbit | 1: 200 |
| β-actin | Servicebio | GB12001 | Mouse | 1: 1000 |
