# Supplementary material for: tRF-Val-CAC-016 modulates the transduction of CACNA1d-mediated MAPK signaling pathways to suppress the proliferation of gastric carcinoma
Source: Cell Commun Signal. 2022 May 19;20:68. doi: 10.1186/s12964-022-00857-9 (PMC9118711; doi:10.1186/s12964-022-00857-9)
Supplement: Supplementary file 4 — Additional file 3.Table S2. The sequence of siRNAs and tRF mimics. [file 12964_2022_857_MOESM4_ESM.docx]

**Table S2 The sequence of siRNAs and tRF mimics.**

| **Names** | **Sequence (5’-3’)** |
| --- | --- |
| tRF-Val-CAC-016 mimics | AAGTGGTTCCCGTTT |
| CACNA1d-F | TTCCCTCAAGCACTTCTCACAG |
| CACNA1d-R | GCCATCGTACATCACAGCATTC |
| si-CACNA1d-1 | CGAAGAGCCTGCATTAGTA |
| si-CACNA1d-2 | GAACGGATCTGGCAAAACA |
| si-CACNA1d-3 | GGGAAGTTCTATCGCTGTA |
| U6-F | CTCGCTTCGGCAGCACA |
| U6-R | AACGCTTCACGAATTTGCGT |
| GAPDH-F | ACCCACTCCTCCACCTTTGAC |
| GAPDH-R | TGTTGCTGTAGCCAAATTCGTT |

**Note** F, forward, R, reverse. The sequence information of all siRNA controls, ASO controls, miRNA primers, and probes is not public according to the declaration of RiboBio (Guangzhou, China).
